# Galvanism

**Published:** 1864-02

**Authors:** 


GALVANISM.—A most effectual manner of arousing the energies of a patient becoming comatose from opium, is the use of galvanism. In a child of seven months, it was found that its application across the cheeks aroused it most. Cold affusion had lost its power.

